# Knowledge of glaucoma and associated factors among adults in Gish Abay town, Northwest Ethiopia

**DOI:** 10.1186/s12886-019-1295-7

**Published:** 2020-01-06

**Authors:** Zewdu Yenegeta, Ayanaw Tsega, Yezinash Addis, Fisseha Admassu

**Affiliations:** 1 Department of Ophthalmology, St. Paul’s Hospital Millennium Medical College, Addis Ababa, Ethiopia; 2 Department of Optometry, College of Medicine and Health Sciences, University of Gondar, Gondar, Ethiopia; 3 Department of Ophthalmology, College of Medicine and Health Sciences, University of Gondar, Gondar, Ethiopia

**Keywords:** Knowledge, Glaucoma, Associated factors, Gish Abay, Ethiopia

## Abstract

**Background:**

Glaucoma is a global public health problem and it is the leading cause of irreversible blindness worldwide. Even though public knowledge of glaucoma is a key for early case identification and prevention of blindness, it is unknown in the study area, making provision of interventions difficult. Therefore, this study was intended to assess knowledge of glaucoma and associated factors among adults in Gish Abay town, Northwest Ethiopia, 2018.

**Methods:**

A Community based cross - sectional design study with systematic random sampling technique was used to select 630 adults. The study was conducted from April to May, 2018. Data was entered into Epi Info version 7 and was analysed by Statistical Package for Social Sciences version 23. Binary logistic regression model and adjusted odds ratio with 95% confidence level was used to identify the significant factors associated with knowledge of glaucoma. Variables with P–value ≤0.05 was considered statistically significant.

**Results:**

About 594 adults were participated with a response rate of 94%. Above half of (52%) the participants were females with median age of 28 years. The proportion of good knowledge was demonstrated in 16.8% [95% CI; 14.0, 19.9]. Educational status: primary education [AOR; 2.89: 1.41, 5.90], secondary education [AOR; 3.03: 1.47, 6.24] college and above [AOR; 5.18: 2.21, 12.13], history of eye examination [AOR; 6.52: 3.37, 12.63]; family history of glaucoma [AOR; 12.08: 4.13, 35.30] and higher income level [AOR; 3.11: 1.55, 6.25] were positively associated with good knowledge of glaucoma.

**Conclusions:**

The proportion of good knowledge of glaucoma was low. Higher educational status, positive family history of glaucoma, eye examination and higher income level were significantly associated with knowledge of glaucoma.

## Background

Glaucoma is a group of disease characterized by progressive optic neuropathy and visual field defect [1]. Fifteen percent’s of worlds blindness is attributed due to glaucoma and around 600,000 people go blind annually [2]. In 2013, the number of people with glaucoma worldwide was estimated to be 64.3 million [3]. This is expected to raise to 76 million in 2020 and 111 million in 2040 [3]. Africa accounts for 15% worlds blindness burden due to glaucoma [3]. In Ethiopia, glaucoma is the fifth common cause of blindness which results an irreversible sight loss for an estimated 62,000 Ethiopians [4, 5].

The increasing prevalence of glaucoma is expected to cause a significant economic burden and poor quality of life [6–8].

High glaucoma morbidity among some African communities may be attributed to low trained professionals, low awareness, under-utilization of eye care service as well as limited availability of treatment procedures [9–11]. It has been estimated that half of glaucoma patients are already blind in at least one eye in presentation in Africa [12]. The situation is worse in sub Saharan Africa where it is further compounded by poor awareness and knowledge of glaucoma in the region [13].

Raising public awareness early and creating knowledge of glaucoma is a key means of addressing its devastating consequences by enhancing people alertness, increasing regularly eye screening practice and increase the chance of identifying undetected cases [5]. Contrary to this, lack of awareness of glaucoma appeared to influence self-care practices [14].

Poor public awareness, knowledge and misconceptions associated with glaucoma is challenge for public health professionals [14].

According to different literatures reviewed educational status [15–17], positive family history of glaucoma [16, 18, 19], history of eye examination [10, 12, 17], type of occupation (medical profession) [10, 20], positive glaucoma status [16], having a chronic disease like diabetes mellitus or hypertension or both [19] and high income level [15, 20–23] were positively associated with knowledge of glaucoma.

It is known that awareness leads to knowledge and knowledge to behavior [24]. However, published evidences indicates that knowledge about glaucoma is limited across the globe [14–16, 24, 25]. Therefore, this study is intended to fill this gap while serving as a baseline information for policy makers to evaluate intervention programs and to design future programs and techniques for effective health education based on identified factors.

## Methods

### Study population and study design

A community based cross-sectional design study was conducted in Gish Abay town. It is found in West Gojjam administrative zone. The capital of Sekela, Gish Abay, located 463 km from Addis Ababa. Gish Abay has 2 Keeble’s with 8883 households. This study was conducted between April to May, 2018. All adults ≥18 years were included in the study.

### Sample size determination

Sample size was determined separately for knowledge and the associated factors to address the objectives. The sample size was determined by single population proportion formula with the assumption of 95% confidence level (Z□/2 = 1.96), 4% maximum allowable error (w), 5% non-response rate. The sample size for knowledge taking the proportion, *p* = 49.6 for good and 50.4 for poor [17] was 630 for both.

### Sampling procedure

Systematic random sampling was used for sampling technique. The sampling fraction was determined by taking the ratio of households to the sample size i.e. 14. Then, households were selected by using systematic random sampling method by sampling fraction of 14 to get one adult from each house hold. Finally, one eligible adult was selected from each household using simple random sampling/ lottery method if more than one adult was living in the house.

### Data collection tool and procedures

The questionnaire was adapted from previous studies [11, 16–19, 24, 26]. It was initially prepared in English, translated into Amharic (local language) by language experts, and re translated to English to check consistency in meaning of words and concepts. Almost all of the questions were closed ended. The questionnaire consists three parts: socio-demographic or background information, questions to measure clinical characteristics and awareness of glaucoma, sources of information and knowledge of glaucoma. The data were collected through face to face interview by trained data collectors.

### Operational definition

Knowledge: Knowledge was determined based on 12 knowledge questionnaires and was graded as its level (good, poor). All of the questionnaires were closed ended with possible definitions, risk factors and treatment options of glaucoma. Each correct response was given one point. The total score was out of 12. A score which were ≥ 50%(≥ 6/12) were recorded as good knowledge. Otherwise it was recorded as having poor knowledge.

### Data management and analysis

After checking for clarity and completeness data were entered into Epi Info 7 and exported into statistical package for social science/SPSS version 23 for analysis. Both descriptive and analytical methods were employed for analysis. The descriptive parts of the data were summarized using measures of central tendency and dispersion.

Associated factors for knowledge of glaucoma were identified using binary logistic regression. Variables were fitted into the model by using the enter method. The Hosmer and Lemeshow model fitness were used to check model fitness. Multi-collinearity between the independent variables were checked by tolerance and variance inflation factor (V.I.F). Adjusted odds ratio with 95% confidence level was used to identify the significant factors. Variables with *p*-value of ≤0.05 was considered statistically significant.

## Results

### Socio-demographic characteristics

A total of 594 adults were participated with a response rate of 94.29%. One hundred forty-five adults were aware of glaucoma and further asked for knowledge questions. The median (IQR) age was 28 years [22–45]. About 308 (51.9%) of the respondents were females. Majority 359 (60.4%) of the participants had no formal education (Table 1).

**Table 1:** Socio-demographic and socio-economic characteristics of the study participants in Gish Abay town, Northwest Ethiopia, Jun 2018 (*n* = 594)

Characteristics	Frequency	Percent
Sex		
Female	308	51.9
Male	286	48.1
Age		
18–22	169	28.5
23–27	144	24.2
28–45	140	23.6
≥ 46	141	23.7
Religion		
Orthodox	586	98.7
Others*	8	1.3
Ethnicity		
Amhara	584	98.3
Oromo	10	1.7
Level of education		
No formal education	359	60.4
Primary education	101	17.0
Secondary education	90	15.2
College and above	44	7.4
Marital status		
Single	275	46.3
Married	288	48.5
Divorced	14	2.4
Widowed	17	2.9
Type of occupation		
Farmer	156	26.3
Government employee	48	8.1
Merchant	185	31.1
House wife	72	12.1
Job seeker	64	10.8
Others**	69	11.6
Monthly income		
≤ 750	298	50.2
751–1300	115	19.4
1301–2000	62	10.4
≥ 2001ETB	119	20

Others* Muslim and Catholic; Others** = students, driver, religious leader

### Knowledge of glaucoma

About 100 of participants had good knowledge of glaucoma. The proportions of good knowledge of glaucoma were 16.8% (14.00, 19.9).

Factors associated with knowledge of glaucoma.

In the multivariable analysis after adjusting for age, sex, ethnicity, type of occupation chronic diseases either DM or HTN: educational status, level of income, history of eye examination at least once in life and positive family history of glaucoma were significantly associated with good knowledge of glaucoma (Table 2).

**Table 2:** Binary logistic regression of factors associated with knowledge of glaucoma among adults in Gish Abay town, Northwest Ethiopia, Jun 2018(*n* = 594)

The level of knowledge of glaucoma				
Variables	Good	Poor	COR (95% CI)	AOR (95% CI)
Age category/years				
18–22	22	147	1.00	1.00
23–27	30	114	1.75 (0.96, 3.2	1.51 (0.75, 3.06)
28–45	32	108	1.97 (1.09, 3.59)	1.74 (0.83, 3.67)
≥ 46	16	125	0.85 (0.43, 1.70)	0.80 (0.31, 2.05)
Sex				
Male	60	226	1.00	1.00
Female	40	268	0.56 (0.36, 0.87)	0.72 (0.42, 1.22)
Educational status				
No formal education	36	323	1.00	1.00
Primary education	24	77	2.79 [1.57, 4.96]	2.89[1.41, 5.90]**
Secondary education	23	67	3.08 [1.72, 5.53]	3.03[1.47, 6.24]**
College and above	17	27	5.64 [2.81, 11.35]	5.18[2.21, 12.13]***
Income level/ETB				
≤ 750	20	278	1.00	1.00
751–1300	29	86	4.68 [2.52, 8.70]	3.18 [1.57, 6.42]**
1301–200	15	47	4.43 [2.12, 9.27]	3.53 [1.54, 8.06]**
≥ 2001	36	83	6.02 [3.31, 10.97]	3.11 [1.55, 6.25]**
Eye examination				
No	63	457	1.00	1.00
Yes	37	37	7.25 [4.28, 12.27]	6.52[3.37, 12.63]***
Chronic diseases				
No	83	468	1.00	1.00
Yes	17	26	3.68 [1.92, 7.09]	1.53[0.62, 3.81]
Family history of glaucoma				
No	83	487	1.00	1.00
Yes	17	7	14.24 [5.73, 35.42]	12.08[4.13, 35.30]***

Where, * *p* ≤ 0.05; ** *p* ≤ 0.01; *** *p* ≤ 0.001; 1.00 = reference; *CI* Confidence Interval, *COR* crude odds ratio, *AOR* adjusted odds ratio

## Discussion

The proportion of good knowledge of glaucoma was 16.8% (14.0, 19.9). This finding is lower than reports from Gondar, Northwest Ethiopia (49.6%) [17]. This might be due to differences in the way we measure knowledge is quite different. In the later study, the level of good knowledge was among aware adults where as in this study it was among all adults participating in the study. This might be also due to the differences in educational status. In the Gondar study about 32.7% had no formal education as compared to 60.2% in this study. Previous studies described educational status as the true predictor of knowledge [15–17]. The present finding is also lower than reports from urban Puducherry, India (34%) [19]. This might be due to the difference in proportion of adults who had history of eye examination (31%) as compared to 12% in this study. Previous studies support history of eye examination were directly associated with good knowledge of glaucoma [10, 12, 17].

However, this finding is better than the results reported from Addis Ababa, Capital of Ethiopia (12.1%) [26], Nigeria, Ebonyi State (6.7%) [18], Nepal (5.5%) [27], (1.18%) [28], urban India, Chennai (0.5%) [16] and rural India (1.89%) [24]. This might be due to educational differences among the study participants: about 14.9% [31] and 12% [21] as compared to 22.6% of adults in this study completed higher education.

On the other hand, the finding of the present study is consistent with reports from Tehran, Iran (19.2%) [25].

In this study, adults with higher educational status were positively associated with good knowledge of glaucoma. This was supported by other studies [15–17]. This might be due to as educational status increases the chance of exposure to health related information transmission increases [19–21, 26].

Adults with positive family history of glaucoma were more likely to had good knowledge of glaucoma. This was supported by different studies [16, 18, 19]. This might be due to the fact that this portion of adults would have personal experience with the challenges of the disease and its treatment options and would help them to acquire knowledge of the condition [18].

Eye examination was another important determinant for knowledge of glaucoma. This was supported with different studies [10, 12, 17]. Higher level of income was positively associated with good knowledge of glaucoma. This was supported with different studies [20–22].

## Conclusions

The proportion of good knowledge of glaucoma was lower. Higher educational status, eye examination at least once in life, positive family history of glaucoma and higher income level were positively associated with good knowledge of glaucoma. This implies that the need for improving this knowledge of glaucoma through community health education programs and also improving regular eye check-up practice to the general population specially to those in the rural parts of the region.

## Data Availability

The dataset used and or analysed during this study are available from a corresponding author on a reasonable request from Mr. Zewdu Yenegeta (contact address: zewduyenegeta@gmail.com).
